# Resina Draconis Reduces Acute Liver Injury and Promotes Liver Regeneration after 2/3 Partial Hepatectomy in Mice

**DOI:** 10.1155/2020/2305784

**Published:** 2020-10-07

**Authors:** Zhi-yong He, Kai-han Lou, Jia-hui Zhao, Ming Zhang, Lan-chun Zhang, Ju Li, Hao-fei Yu, Rong-ping Zhang, Hu Wei-yan

**Affiliations:** ^1^The Key Laboratory of Stem Cell and Regenerative Medicine, Institute of Molecular and Clinical Medicine, Kunming Medical University, Kunming 650500, China; ^2^Monash Immune Regeneration and Neuroscience Laboratories, Department of Anatomy and Developmental Biology, Monash University, Clayton, Melbourne 3800, Australia; ^3^School of Pharmaceutical Science & Yunnan Key Laboratory of Pharmacology for Natural Products, Kunming Medical University, Kunming 650500, China; ^4^Yunnan University of Chinese Medicine, Kunming 650500, China

## Abstract

**Aim:**

To investigate the protective effects and possible mechanisms of action of resina draconis (RD) on acute liver injury and liver regeneration after 2/3 partial hepatectomy (PH) in mice.

**Methods:**

2/3 PH was used to induce acute liver injury. Mice were divided into three groups: sham, vehicle + 2/3 PH, and RD + 2/3 PH. Resina draconis was administered intragastrically after 2/3 PH into the RD + 2/3 PH group, and the same volume of vehicle (1% sodium carboxymethyl cellulose) was injected into the vehicle + 2/3 PH group and sham group mice. The index of liver to body weight (ILBW) and proliferating cell nuclear antigen (PCNA) were assayed to evaluate liver regeneration. Blood and liver tissues were collected for serological and western blotting analysis.

**Results:**

Resina draconis protected against 2/3 PH-induced acute severe liver injury and promoted liver regeneration as shown by significantly increased ILBW compared with that of controls. 2/3 PH increased serum AST and ALT levels, which were significantly decreased by RD treatment, while 2/3 PH decreased serum TP and ALB, which were increased by RD treatment. In the RD + 2/3 PH group, PCNA expression was significantly increased compared with the 2/3 PH group. Further, hepatocyte growth factor (HGF), TNF*α*, and EGFR levels were increased in the RD group at postoperative days 2 and 4 compared with the those in the 2/3 PH group.

**Conclusion:**

Our results suggest that RD ameliorates acute hepatic injury and promotes liver cell proliferation, liver weight restoration, and liver function after 2/3 PH, probably via HGF, TNF*α*, and EGFR signaling.

## 1. Introduction

The liver plays a central role in detoxification and protein synthesis and produces digestive biochemicals. It also possesses the unique ability to regenerate after surgical resection or injury to maintain these essential functions [1–4]. The clinical application of partial hepatectomy (PH) or living donor liver transplantation (LDLT) for the treatment of primary or metastatic liver tumors and end-stage liver disease depends on effective liver regeneration [5–7]. Optimizing liver regeneration would have great clinical potential in improving outcomes from liver disease. Two-thirds (2/3) PH is a well-validated experimental model of liver regeneration that has been extensively used to study the underlying molecular and cellular mechanisms [8–12].

Growth factors such as TGF*α*, EGF, and HGF are highly expressed after PH and are thought to be important in regulating hepatocyte activation, proliferation, migration, and survival during liver regeneration [13–15]. Hepatocyte growth factor (HGF) is the major hepatocyte mitogen [16], and HGF is upregulated after PH. HGF initiates hepatocyte DNA synthesis and stimulates hepatocyte growth [16, 17]. Overexpression of HGF also increases hepatocyte proliferation and accelerates liver regeneration after PH [18]. Mice lacking c-MET, the HGF receptor, show delayed liver regeneration after PH, with hepatocytes showing S-phase entry defects [19].

TNF*α* is an important regulator of the “priming phase” of liver regeneration, and blocking TNF*α* signaling in rats prior to PH significantly reduces the proliferation of hepatocytes and nonparenchymal liver cells [20, 21]. TNF*α*, together with its downstream effector IL-6, initiates hepatocyte transition from G0 to G1 phase of the cell cycle [21–23]. TNF*α* also activates transcription factors like STAT3 and NF-*κ*B, which in turn promote the expression of cell cycle regulator genes such as cyclin D [24, 25].

The EGF receptor (EGFR) also plays a vital role in liver regeneration, since liver regeneration is not impaired in mice lacking TGF*α* because other EGFR ligands compensate for the absence of TGF*α* [14, 26]. EGFR is expressed at high levels in the adult liver and regulates liver development, function, and regeneration [13]. Transgenic mice lacking EGFR have a higher mortality rate after PH, with surviving mice showing increased hepatocyte proliferation due to low cyclin D1 expression. The EGFR pathway regulates hepatocyte proliferation and efficient liver regeneration after PH [27, 28].

Resina draconis (RD), a bright resin extracted from *Dracaena cochinchinensis* (Lour.) S. C. Chen, is a Chinese Herbal medicine with a well-established safety and bioactivity record [29, 30]. RD contributes to skin repair and can promote the development of transplanted epidermis, enhance the capillary proliferation [31], and promote the growth of fetal rat skin fibroblasts [32]. It can also improve wound healing [33] and has been used for the treatment of skin diseases [34, 35]. Further, RD can facilitate blood circulation and improve platelet function [36, 37]. RD has anti-inflammatory effects [38] and antitumor activities [39, 40]. Of these multiple activities, RD strongly promotes tissue recovery after injury. However, the effects of RD on liver regeneration after extended hepatectomy have not been investigated. Here, we explored the effect and possible mechanisms of RD treatment on liver regeneration in 2/3 PH mice. The involvement of HGF, TNF*α*, and EGFR signaling in RD-stimulated liver regeneration was also investigated.

## 2. Materials and Methods

### 2.1. Preparation of Drugs

Resina draconis powder (Xishuangbanna Rainforest Pharmaceutical Co., Ltd., Jinghong, China) was dispersed in 1% sodium carboxymethyl cellulose (Na-CMC) at 10 mg/mL, 20 mg/mL, and 40 mg/mL before intragastric injection into mice.

### 2.2. Animals

One-hundred forty-four 6-8 week-old male C57BL/6 mice weighing between 20 and 25 g were used. Animals were divided into three groups (*n* = 8 in each group): sham, vehicle + 2/3 PH, and RD + 2/3 PH. The RD + 2/3 PH group received intragastric injections of 0.2 mL RD solution at doses of 0.1 g/kg, 0.2 g/kg, or 0.4 g/kg body weight once daily for 10 days after 2/3 PH surgery. All experiments were conducted under the institutional guidelines of the Animal Ethics Committee, Kunming Medical University.

### 2.3. Surgical Procedure and Anesthesia

2/3 PH was performed in the vehicle + 2/3 PH and RD + 2/3 PH groups using a slight modification of the methods developed by Harkness [41, 42]. Briefly, after general anesthesia, the left lateral, left median, and right median lobes were resected. Laparotomy was performed in the sham group without ligation and 2/3 PH.

### 2.4. Liver Tissue Collection

On days 0, 2, 4, 6, 8, and 10 after 2/3 PH, animals in each group were weighed and sacrificed humanly, liver tissues were collected and weighed, and the index of liver to body weight (ILBW) recorded.

### 2.5. Serum Parameters

Peripheral blood was collected and centrifuged. Serum alanine transaminase (ALT), aspartate transaminase (AST), alkaline phosphatase (ALP), albumin (ALB), and total protein (TP) were measured in U/L with a serum multiple biochemical analyzer (Diamond Diagnostics Inc., Holliston, USA).

### 2.6. Western Blotting

Liver tissue proteins were prepared as previously described [43]. Western blotting was performed using antibodies targeting PCNA (Invitrogen, Thermo Fisher Scientific, Waltham, MA, USA), *β*-actin (Sigma Aldrich, St. Louis, MO, USA), HGF (Abcam, Cambridge, UK), EGFR (Abcam), and TNF-*α* (Abcam). Super Signal West Dura Extended Duration Substrate (Pierce, Thermo Fisher Scientific, Waltham, MA, USA) was used to visualize antibody-antigen complexes.

### 2.7. Data Analysis

Three-way ANOVA was applied to analyze BW, ILBW, TP, ALB, ALT, ALP, and AST using the factors 2/3 PH operation, RD treatment, and time after injury. Tukey's post hoc tests were applied as appropriate. Student's *t* test was used to analyze data between two groups. All values were expressed as mean ± SEM, and a *p* value <0.05 was considered statistically significant.

## 3. Results

### 3.1. Resina Draconis Treatment Promotes Liver Weight Recovery after 2/3 PH

**Figure 1:**
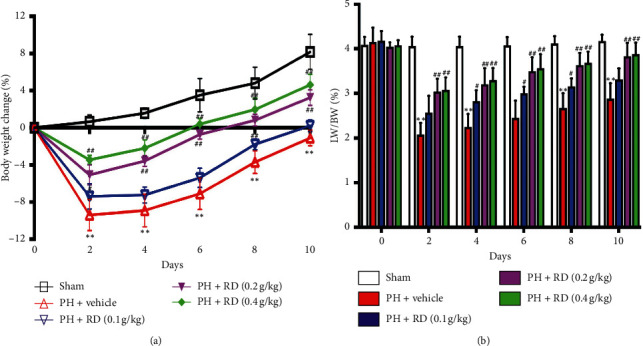
Resina draconis increases body weight and index of liver to body weight (ILBW) after 2/3 PH. (a) RD treatment at different doses increases body weight gain over time in mice receiving 2/3 PH. Results are represented as mean ± SEM of percentage change in body weight versus original body weight before 2/3 PH. *n* = 8 mice/time point. ^*∗∗*^*p* < 0.01, vehicle + 2/3 PH group *vs* sham group; ^##^*p* < 0.01, RD + 2/3 PH group *vs* vehicle + 2/3 PH group; ^#^*p* < 0.05, RD vehicle + group *vs* vehicle + 2/3 PH group. (b) RD treatment increases ILBW over time in mice receiving 2/3 PH. Data are expressed as mean ± SEM. *n* = 8 mice/time point. ^*∗∗*^*p* < 0.01, vehicle + 2/3 PH group *vs* sham group; ^##^*p* < 0.01, RD + 2/3 PH group *vs* vehicle + 2/3 PH group; ^#^*p* < 0.05, RD + 2/3 PH group *vs* vehicle + 2/3 PH group.

To address the effect of RD on general mouse health after PH, mouse body weights were monitored after 2/3 PH. The body weights of mice reduced on days 2 and 4 after 2/3 PH followed by weight gain on day 6. RD treatment at different doses induced faster and longer-lasting weight gain than vehicle group over 10 days (Figure 1(a)).

The ILBW from days 2 to 10 was measured after 2/3 PH to address the effect of RD on liver recovery. The ILBW was significantly higher on days 2, 4, 6, 8, and 10 in RD-treated mice versus vehicle controls (Figure 1(b)). Of the different RD concentrations tested, the 0.2 g/kg and 0.4 g/kg doses were most effective, with no significant difference between the 0.4 g/kg treatment and 0.2 g/kg treatment. Therefore, 0.2 g/kg was used in subsequent experiments. These data indicate that RD treatment promotes liver recovery.

Resina draconis treatment prevents decreases in serum total protein (TP) and albumin (ALB) after 2/3 PH.

**Figure 2:**
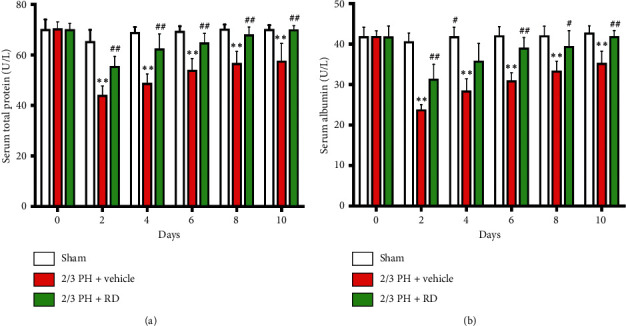
Resina draconis treatment prevents loss of serum TP and ALB after 2/3 PH. (a) RD treatment increases serum TP over time in mice receiving 2/3 PH. Results are represented as mean ± SEM. *n* = 8 mice/time point. ^*∗∗*^*p* < 0.01, vehicle + 2/3 PH group *vs* sham group; ^##^*p* < 0.01, RD + 2/3 PH group *vs* vehicle + 2/3 PH group. (b) RD treatment increases serum ALB over time in mice receiving 2/3 PH. Data are expressed as mean ± SEM. *n* = 8 mice/time point. ^*∗∗*^*p* < 0.01, vehicle + 2/3 PH group *vs* sham group; ^##^*p* < 0.01, RD + 2/3 PH group *vs* vehicle + 2/3 PH group; ^#^*p* < 0.05, RD group *vs* 2/3 PH group.

Given that RD treatment promoted liver regeneration, we further measured changes in serum total protein (TP) and albumin (ALB). TP and ALB levels were attenuated in both the 2/3 PH group and the RD-treated 2/3 PH group on days 2, 4, 6, 8, and 10 after PH. However, TP and ALB levels were significantly higher in the RD-treated 2/3 PH group compared with the vehicle + 2/3 PH group (*p* < 0.01) (Figures 2(a) and 2(b)). Furthermore, there was no unequivocal changes in the RD-treated 2/3 PH group and the sham group on days 4, 6, 8, and 10 after PH.

### 3.2. Resina Draconis Treatment Restores Liver Function after 2/3 PH

**Figure 3:**
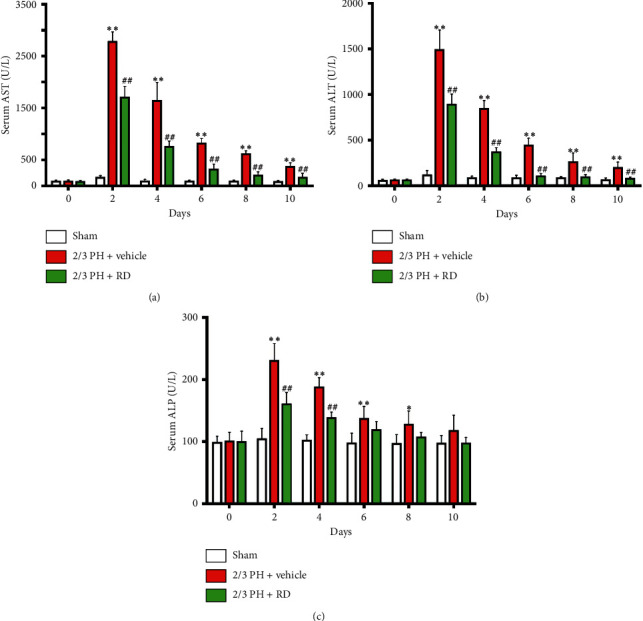
Resina draconis treatment prevents loss of liver function after 2/3 PH. (a) Serum AST levels were significantly increased in both the 2/3 PH group and the RD group on days 2, 4, 6, 8, and 10 following 2/3 PH. Results are represented as mean ± SEM. *n* = 8 mice/time point. ^*∗∗*^*p* < 0.01, vehicle + 2/3 PH group *vs* sham group; ^##^*p* < 0.01, RD + 2/3 PH group *vs* vehicle + 2/3 PH group. (b) Serum ALT levels were significantly increased in both the 2/3 PH group and the RD group on days 2, 4, 6, 8, and 10 following 2/3 PH. Results are represented as mean ± SEM. *n* = 8 mice/time point. ^*∗∗*^*p* < 0.01, vehicle + 2/3 PH group *vs* sham group; ^##^*p* < 0.01, RD + PH group *vs* vehicle + 2/3 PH group. (c) Serum ALP levels were significantly increased in both the 2/3 PH group and the RD group on day 2, 4, 6, and 8 following 2/3 PH. Results are represented as mean ± SEM. *n* = 8 mice/time point. ^*∗∗*^*p* < 0.01, vehicle + 2/3 PH group *vs* sham group; ^*∗*^*p* < 0.05, vehicle + 2/3 PH group *vs* sham group; ^##^*p* < 0.01, RD + 2/3 PH group *vs* vehicle + 2/3 PH group.

Serum AST, ALT, and ALP levels are biomarkers of liver function. Serum AST and ALT levels significantly increased in both the 2/3 PH and RD groups on days 2, 4, 6, 8, and 10 after PH. However, the levels were significantly different in the 2/3 PH group and the RD group (*p* < 0.01) (Figures 3(a) and 3(b)). ALP levels also significantly increased in the 2/3 PH and RD groups on days 2, 4, 6, and 8 after PH. However, the levels were significantly different in the 2/3 PH group and the RD group on days 2 and 4 (*p* < 0.01) (Figure 3(c)). Interestingly, the ALP and ALT levels in the RD group and vehicle group showed no significant difference on days 6, 8, and 10 after PH. These results indicate that RD treatment not only enhances liver regeneration after 2/3 PH but also restores 2/3 PH-induced loss of liver function as measured by liver function biomarkers.

### 3.3. Resina Draconis Treatment Enhances Hepatocyte Proliferation after 2/3 PH

**Figure 4:**
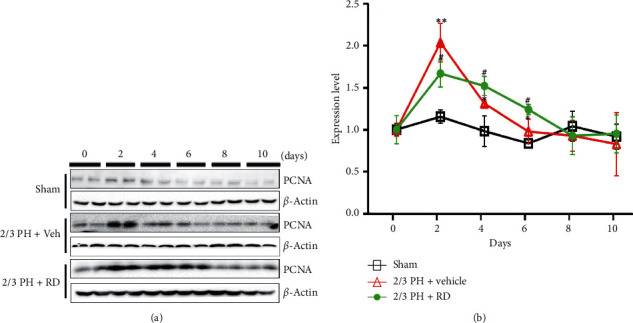
Resina draconis treatment enhances hepatocyte proliferation after 2/3 PH. (a). Representative western blotting analysis of PCNA protein in mouse livers after 2/3 PH and sham operation. RD treatment increases PCNA expression after 2/3 PH. (b). Quantification of PCNA normalized to *β*-actin. Data are expressed as mean ± SEM. *n* = 5. ^*∗∗*^*p* < 0.01, 2/3 PH group *vs* sham group; ^*∗*^*p* < 0.05, 2/3 PH group *vs* sham group; ^##^*p* < 0.01, RD + 2/3 PH group *vs* vehicle + 2/3 PH group; ^#^*p* < 0.05, RD + 2/3 PH group *vs* vehicle + 2/3 PH group.

Liver regeneration is closely related to hepatocyte proliferation. As RD promoted liver recovery, we hypothesized that RD enhanced hepatocyte proliferation. To address this hypothesis, we compared proliferating cell nuclear antigen (PCNA) levels in liver tissues between the different groups. PCNA expression was significantly higher on days 2, 4, and 6 in the RD-treated group compared with the 2/3 PH group (Figures 4(a) and 4(b)), with no observable change in PCNA expressed observed in the sham group.

### 3.4. Resina Draconis Treatment Induces HGF and Increases EGFR and TNF*α* after 2/3 PH

**Figure 5:**
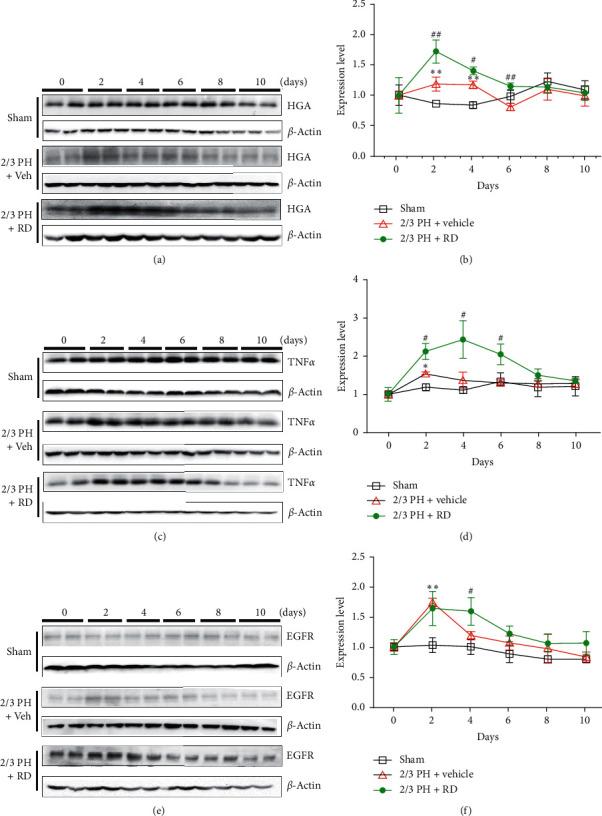
Resina draconis treatment affects HGF, EGFR, and TNF*α* levels after 2/3 PH. (a) and (b). Representative western blotting analysis of HGF protein in mouse livers after 2/3 PH and sham operations. RD treatment increases the HGF expression after 2/3 PH. Quantifications were normalized to *β*-actin. (c) and (d). Representative western blotting analysis of HGF protein in mouse livers after 2/3 PH and sham operations. RD treatment increases TNF*α* expression after 2/3 PH. Quantifications were normalized to *β*-actin. (e) and (f). Representative western blotting analysis of EGFR protein in mouse livers after 2/3 PH and sham operations. RD treatment increases the TNF*α* expression after 2/3 PH. Quantifications were normalized to *β*-actin. All the data are expressed as mean ± SEM. *n* = 5. ^*∗∗*^*p* < 0.01, vehicle + 2/3 PH group *vs* sham group; ^*∗*^*p* < 0.05, vehicle + 2/3 PH group *vs* sham group; ^#^*p* < 0.05, RD + 2/3 PH group vs 2/3 PH group.

HGF, EGFR, and TNF*α* are important growth factors that stimulate hepatocyte mitogenesis in liver regeneration [44, 45]. To explore the possible mechanism by which RD promotes liver regeneration after 2/3 PH, HGF, EGFR, and TNF*α* expressions were measured in liver tissue by western blotting. In the vehicle + 2/3 PH group and RD + 2/3 vehicle group, HGF, EGFR, and TNF*α* increased significantly on day 2 after 2/3 PH and decreased progressively to preoperative levels by the end of the observation period. However, the levels of HGF (Figures 5(a) and 5(b)), EGFR (Figures 5(c) and 5(d)), and TNF*α* (Figures 5(e) and 5(f)) were significantly higher on day 2 and day 4 after 2/3 PH (*p* < 0.05) in the RD + 2/3 PH group than in vehicle + 2/3 PH group and sham group. Further, trends in these increases continued to day 6 and day 8.

## 4. Discussion

Despite clinical advances, liver diseases remain a severe health problem worldwide. Liver resection is the primary treatment modality for liver cancer and other serious liver diseases [46–52]. Liver regeneration plays a vital role in recovery after resection. Acute liver failure after hepatectomy is a main cause of postoperative mortality and morbidity [53–57]. How to protect against postoperative liver injury and promote liver regeneration remains an urgent unmet clinical need. RD has been used for many years to promote wound healing [32, 58] and as an anticoagulant [59–61]. Several studies have shown that RD has hemostatic and anti-inflammatory properties and promotes blood circulation and skin repair [62–65]. Here, for the first time, we provide evidence that RD treatment is beneficial to liver regeneration and protects against liver injury after 2/3 PH. Furthermore, RD-stimulated HGF, EGFR, and TNF*α* signaling during liver regeneration after 2/3 PH. These findings offer a new avenue for protecting against postoperative liver failure and controlling liver regeneration after liver resection.

The exact mechanisms of liver regeneration after 2/3 PH are complicated and have not been fully defined, although a number of growth factors are implicated in the process. HGF, EGFR, and TNF*α* are potential initiators of liver regeneration and increase immediately and are maintained after 2/3 PH. Here, we found that the expressions of HGF, EGFR, and TNF*α* increased significantly after 2/3 PH compared with those of controls on days 2 and 4 after 2/3 PH and were significantly higher in the RD-treated 2/3 PH group than in the vehicle-treated 2/3 PH group. Expression of these growth factors is a key step to initial hepatocyte proliferation and improving liver regeneration and functional recovery. The promitogenic effect of RD-induced expression of these factors was further supported by the observation of higher expression of proliferating cell nuclear antigen (PCNA) in RD-treated mice.

Serum AST and ALT liver function biomarkers are inversely related to the severity of liver damage [66–68]. We found that both ALT and AST levels were significantly higher in the 2/3 PH group mice than in the sham group mice on day 2 and day 4 after 2/3 PH and that RD treatment reversed these changes. TB and ALB are markers of liver synthetic function [69–72]. Our study revealed that TB and ALB levels were higher in the RD-treated 2/3 PH group on day 2 and day 4 after the operation than in vehicle-treated 2/3 PH. Interestingly, RD-treated not only reduced the liver function biomarker levels compared with the vehicle-treated PH group but also it recovered levels to sham over the study period. These results strongly indicate that RD treatment protects against acute liver damage and stimulates liver regeneration after 2/3 PH.

Taken together, our results confirm that intragastric injection of RD after 2/3 PH prevents acute liver failure and promotes liver regeneration. HGF, EGFR, and TNF*α* are involved in this process. These results indicate that RD may be useful in the treatment of acute liver injury.

## Data Availability

The data used to support the study findings are available within the article.

## Abbreviations

RD: Resina draconis
PH: Partial hepatectomy
ILBW: Index of liver to body weight
PCNA: Proliferating cell nuclear antigen
AST: Aspartate transaminase
ALT: Alanine transaminase
TP: Total protein
ALB: Albumin
HGF: Hepatocyte growth factor
TNF*α*: Tumor necrosis factor
EGF: Epidermal growth factor
LDLT: Living donor liver transplantation
NF-*κ*B: Nuclear factor kappa B.

## Supplementary Materials

Supplementary Materials This section includes graphical abstract.
