# Supplementary figures and images for: Resina Draconis Reduces Acute Liver Injury and Promotes Liver Regeneration after 2/3 Partial Hepatectomy in Mice
Source: Evid Based Complement Alternat Med. 2020 Oct 7;2020:2305784. doi: 10.1155/2020/2305784 (PMC7563078; doi:10.1155/2020/2305784)

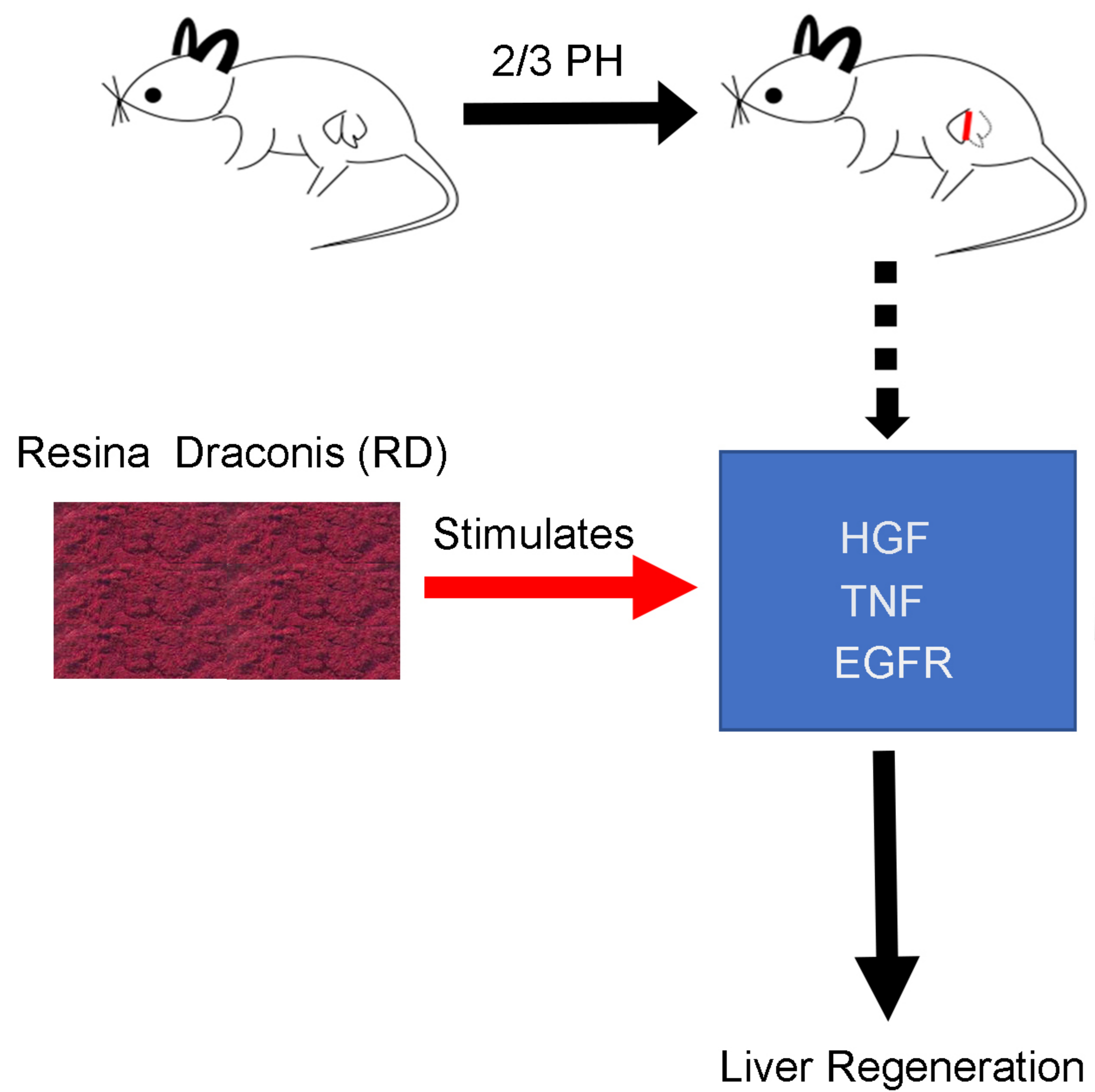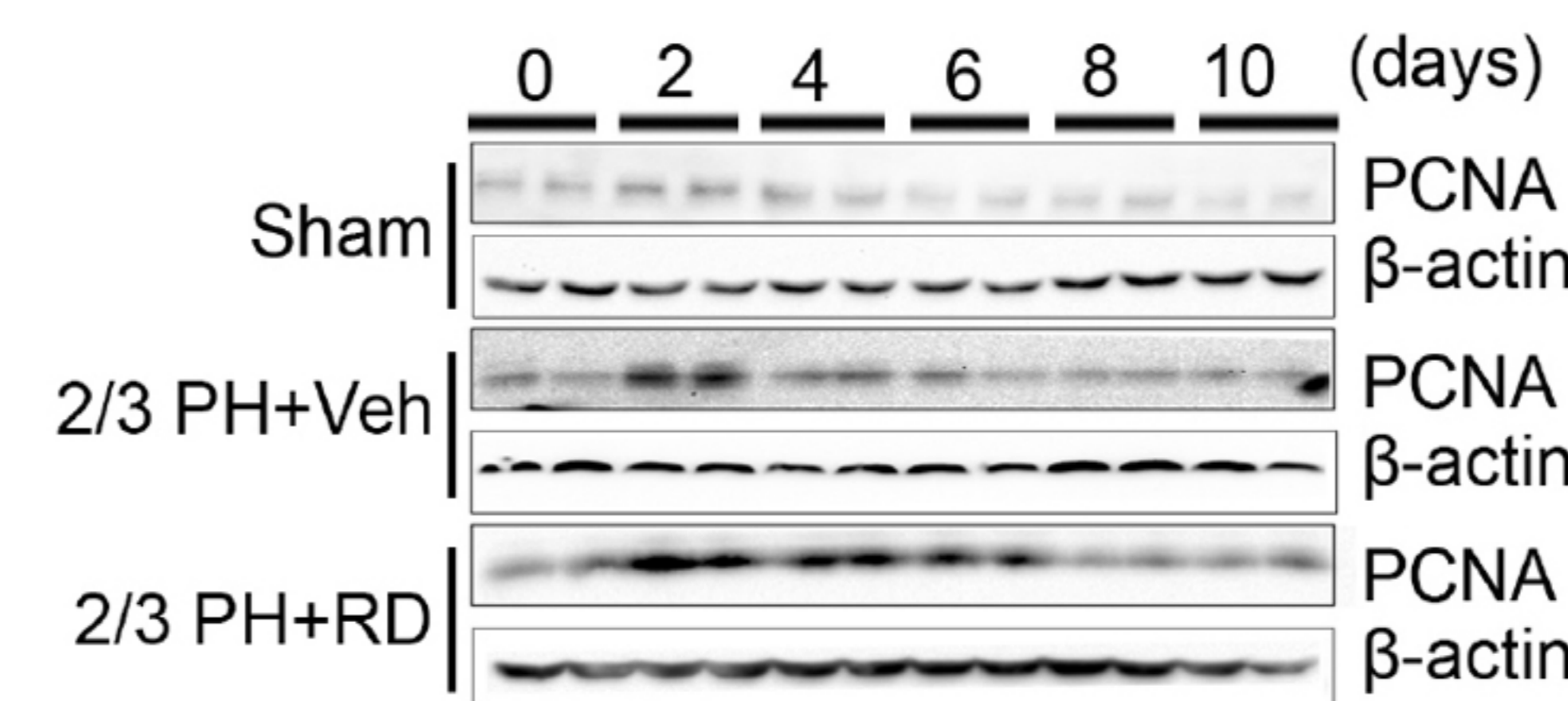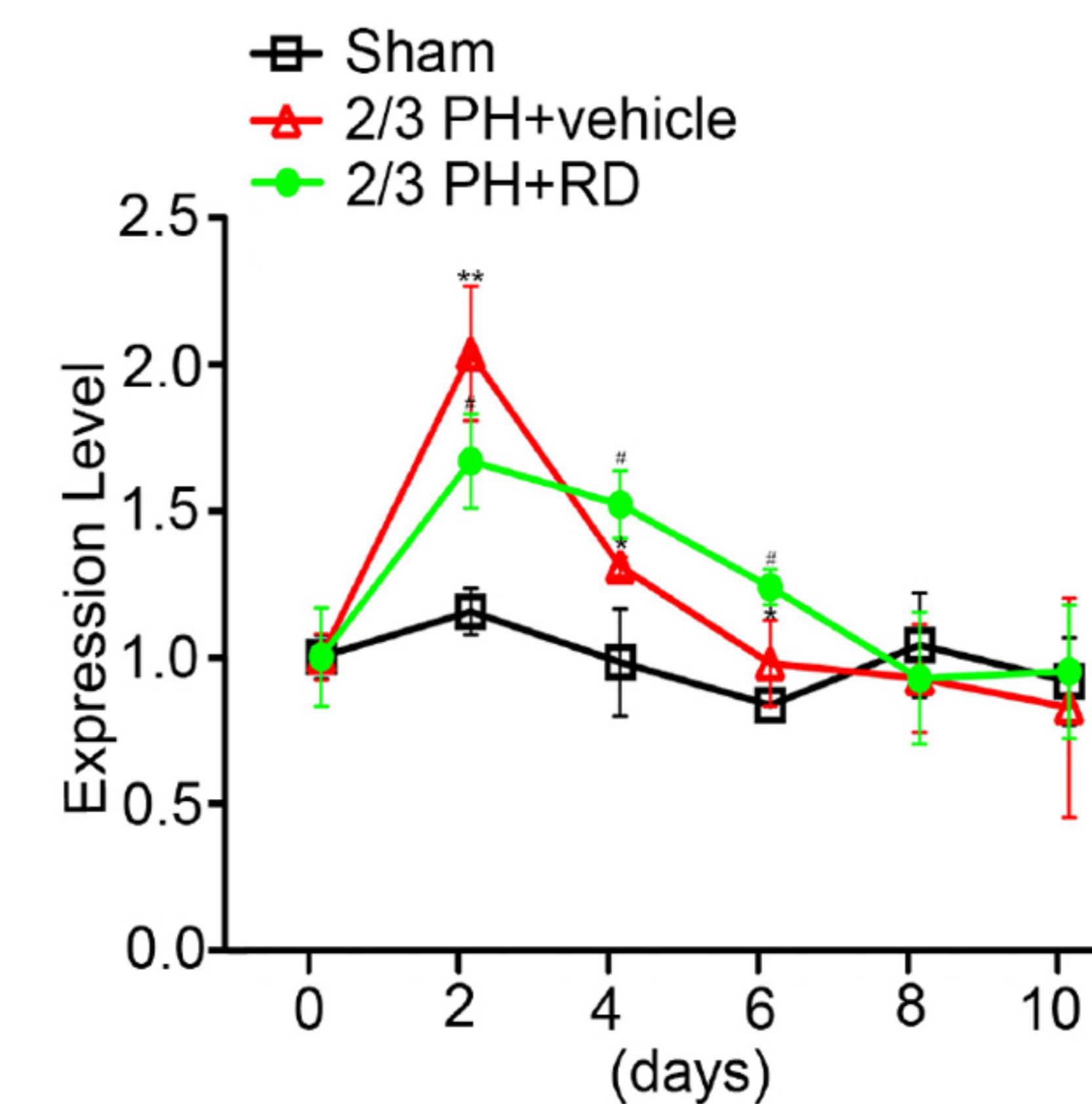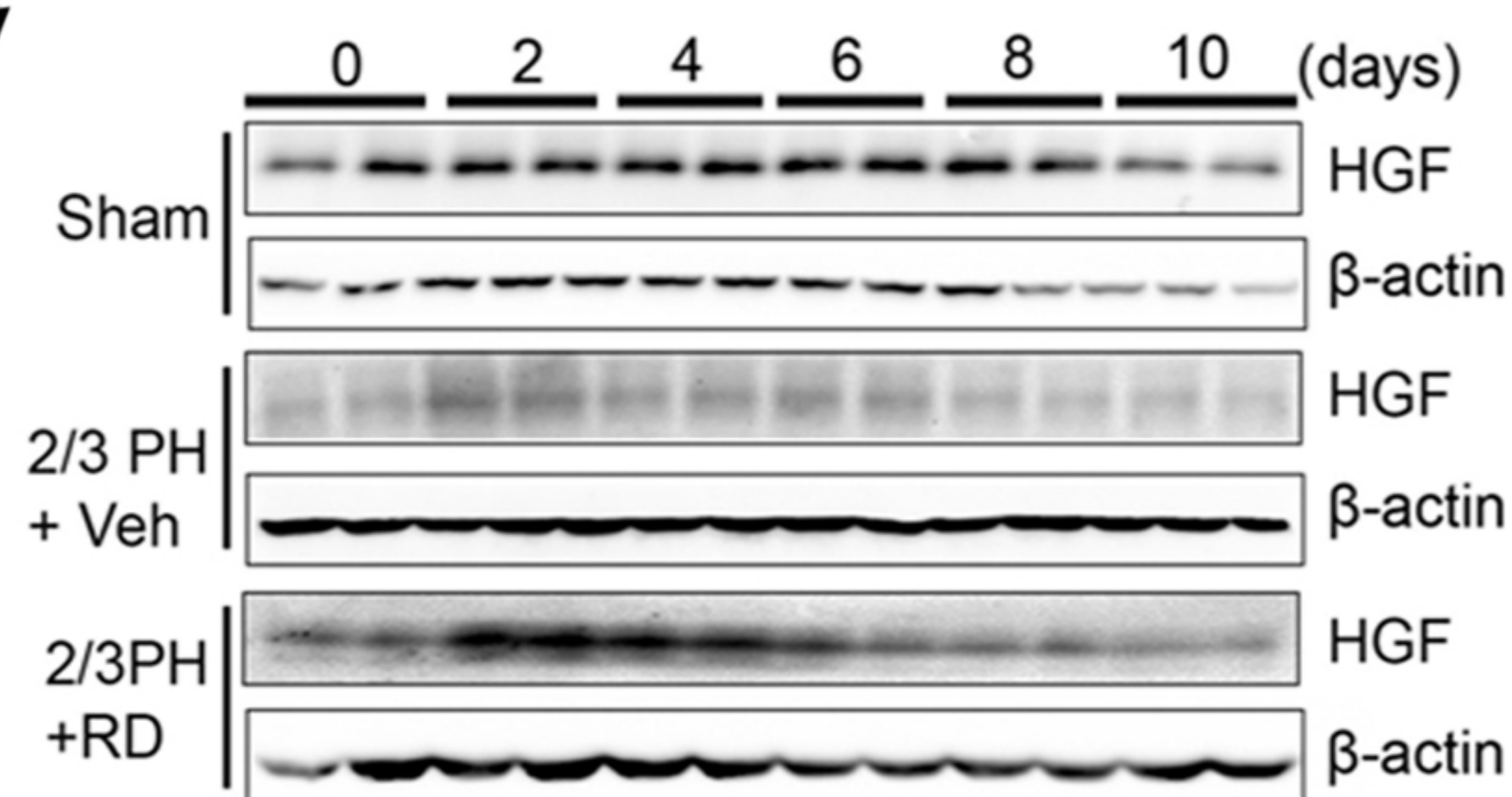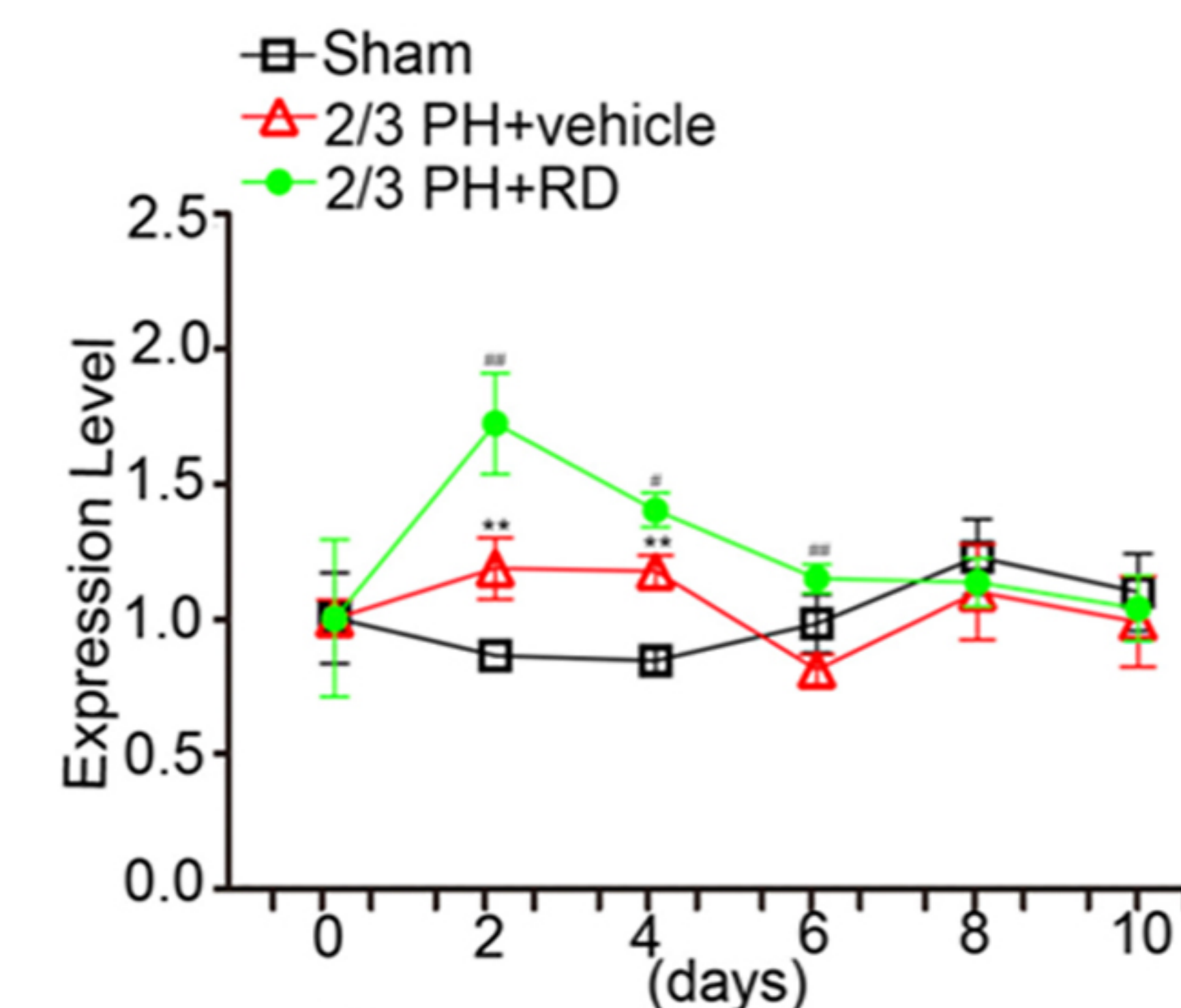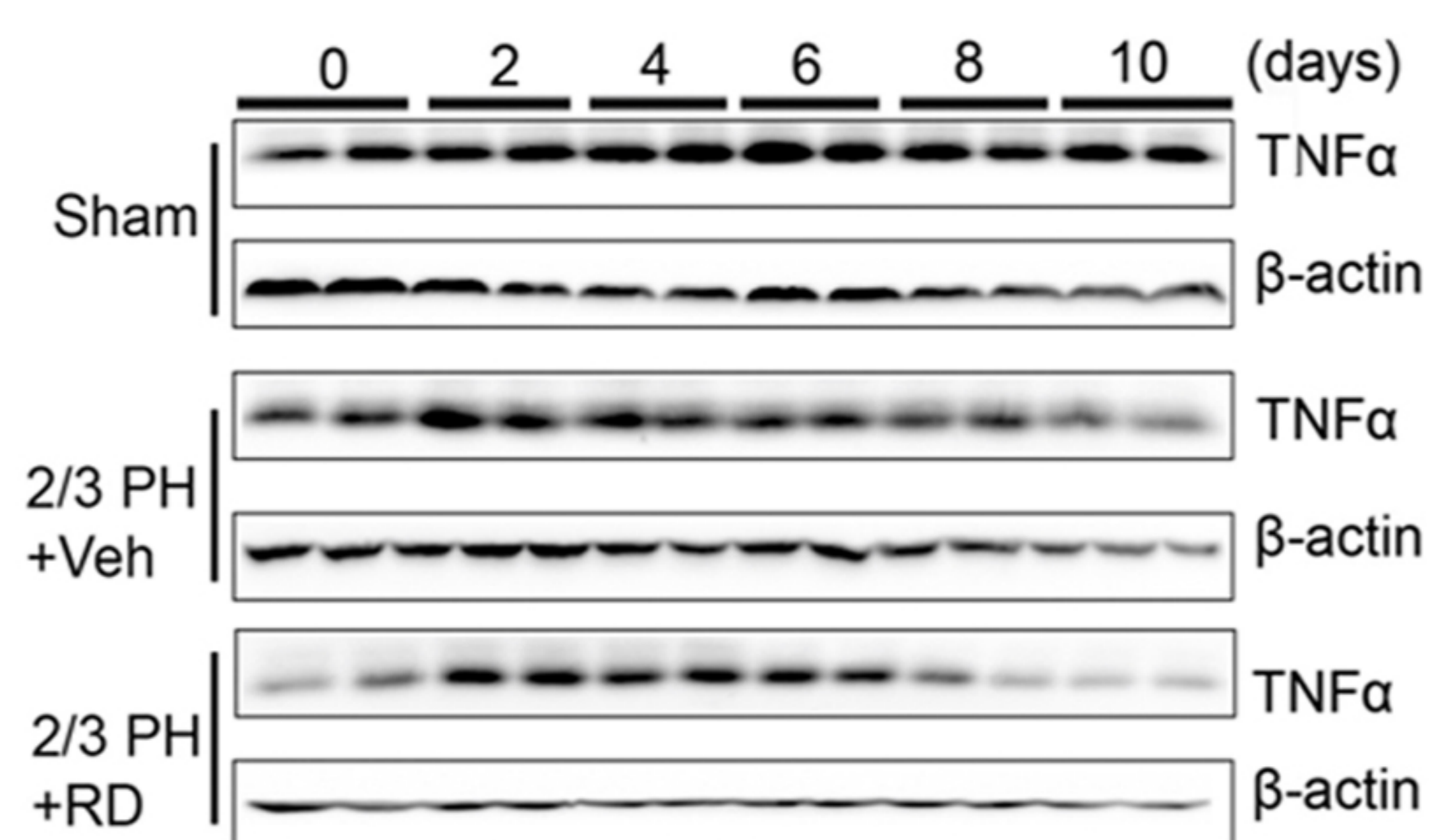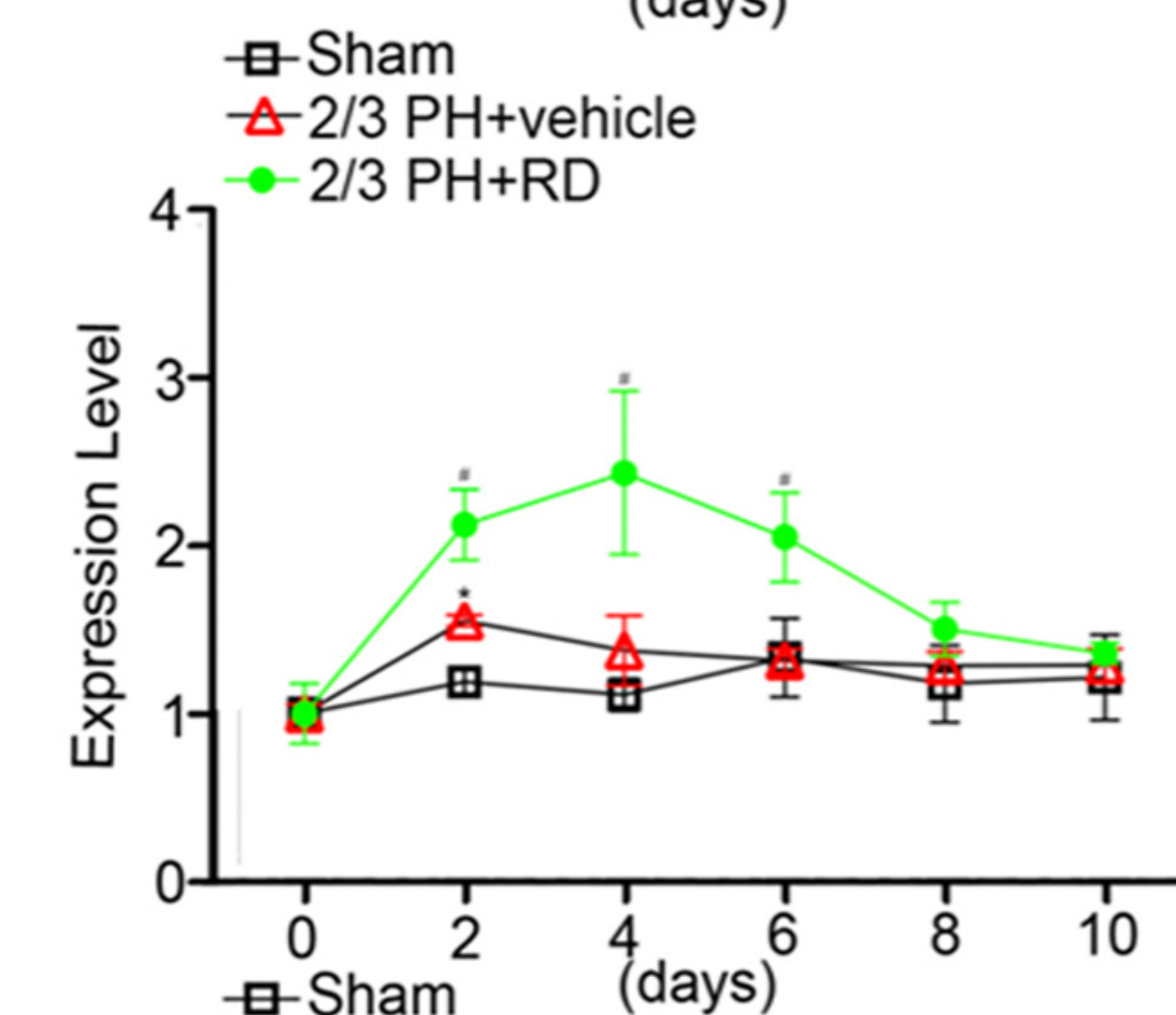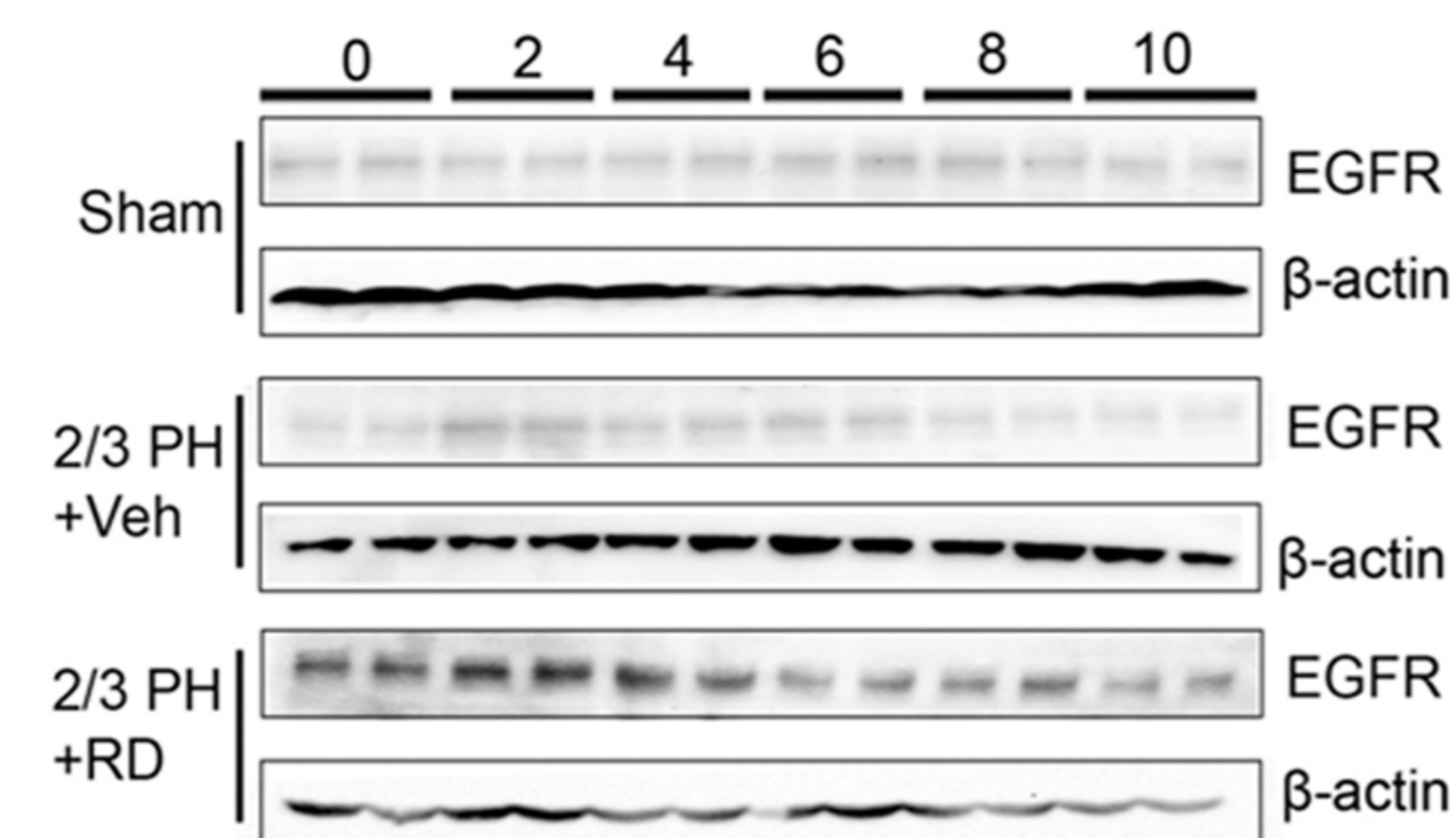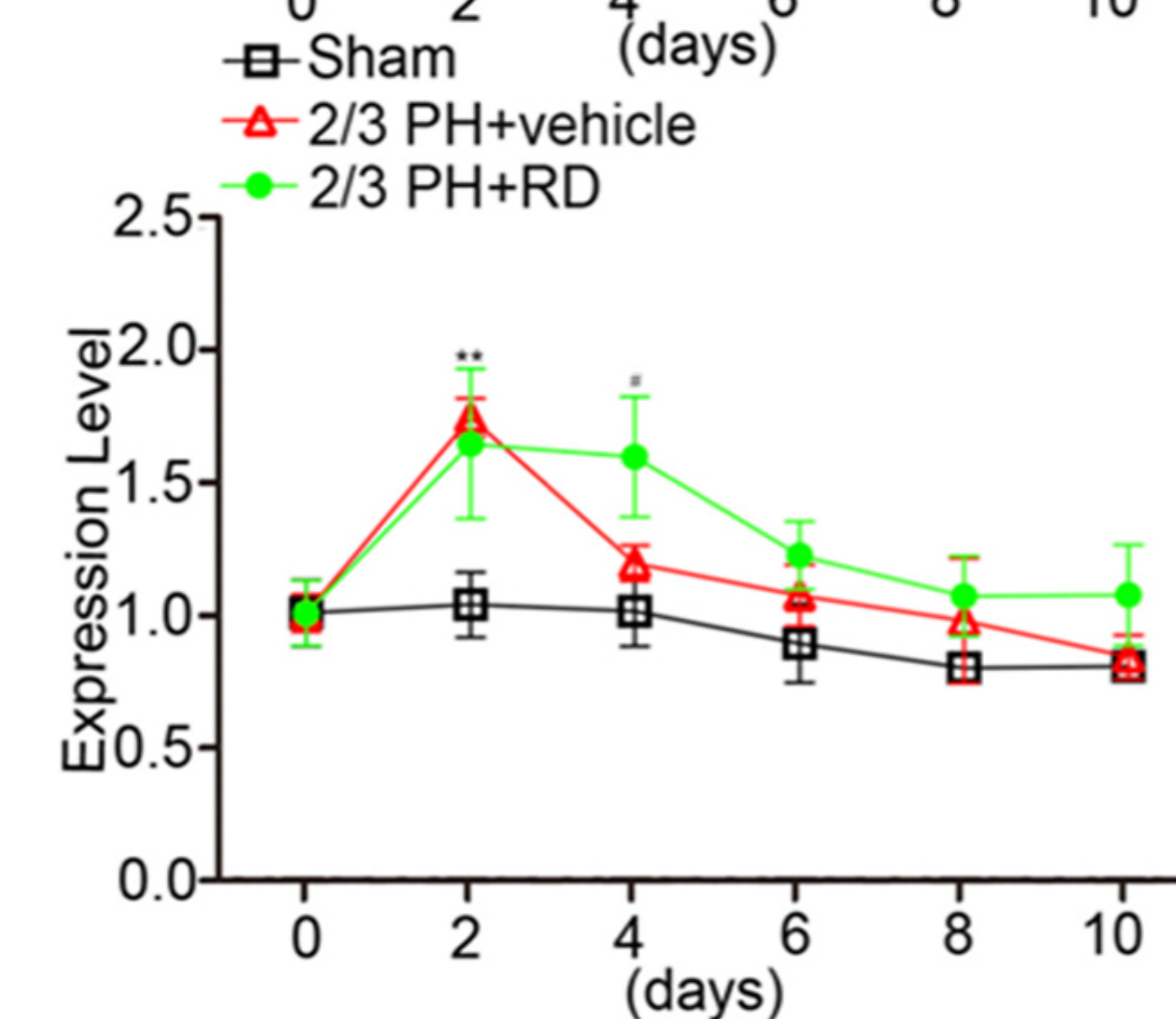

Supplement: Supplementary Materials — This section includes graphical abstract. [file 2305784.f1.pdf]
